# World Allergy Organization Journal (WAO Journal) at five years: updates and changes

**DOI:** 10.1186/1939-4551-6-1

**Published:** 2013-01-31

**Authors:** Lanny J Rosenwasser

**Affiliations:** 1 Allergy & Immunology Division, Children’s Mercy Hospital & Clinics, University of Missouri – Kansas City School of Medicine, 2401 Gillham Road, Kansas City, Missouri 64108, USA

## 

The *World Allergy Organization Journal (WAO Journal)* has recently published a number of position papers, statements and guidelines on topics of global importance in allergy and clinical immunology, including hereditary angioedema [1], urticaria and angioedema [2], probiotics in pediatrics [3], biodiversity and allergic disease [4], as well as the latest ICON, or International Consensus document, on eosinophil disorders [5], an effort that the World Allergy Organization (WAO) led as a participating organization in the International Collaboration in Asthma, Allergy and Immunology (iCAALL). These key articles and more are free for download in Volume 5 of the Journal (http://www.waojournal.org). This significant publishing activity represents the ongoing goals of the *WAO Journal* to provide the most up-to-date and useful information on critical topics of global relevance in the field of allergy/immunology. Since the switch by the World Allergy Organization (WAO) of its Journal to an online-only format five years ago, the Journal has disseminated timely, informative original studies and reviews that offer a global perspective. Now in the sixth year of publication, there are a number of important updates and changes regarding the *WAO Journal* that should be highlighted.

The *WAO Journal* is now published by BioMed Central, as of January 2013, introducing a new era for the Journal in the open-access publishing arena. The Journal’s former publisher, Lippincott Williams & Wilkins, partnered with WAO to create and develop the *WAO Journal* as a fully online publication, and at the time of its launch, it was one of the few of its kind. This recent move to open access publishing reinforces the vision of the *WAO Journal* as a fully accessible authoritative resource for allergists, immunologists and all medical professionals interested in the field. While the Journal has been freely available to all individual members of the WAO Member Societies, it is now fully open to readers everywhere, and its contents will also be available through PubMed Central. This move is pmount in the continuing evolution of the *WAO Journal* as an indispensable source of new research and authoritative review of scientific and clinical advances and knowledge in the allergy community worldwide.

Another significant change coming in the Journal’s sixth year is an editorial transition, as this will be my last year as Editor-in-Chief. This represents a completion of my five-year tenure in this role. Over the next six months WAO will be searching for and evaluating a potential new Editor-in-Chief, derived and solicited from all corners of our community. The new Editor-in-Chief will ideally serve for a few months as Deputy Editor-in-Chief, assuming full responsibility as Editor-in-Chief in January 2014. It is satisfying to see, during my term as Editor-in-Chief, the progress the Journal has made as the flagship publication of the World Allergy Organization in providing a global forum for the exchange of research and information on allergy, asthma, and clinical immunology through publication of original research, clinical reviews, position papers, and epidemiological studies that contribute practice-relevant science in patient care.
